# Comparison of Anti-Microbial Effects of Low-Level Laser Irradiation and Microwave Diathermy on Gram-Positive and Gram-Negative Bacteria in an In Vitro Model

**DOI:** 10.3390/medicina55070330

**Published:** 2019-07-02

**Authors:** Snehil Dixit, Irfan Ahmad, Abdulrahim Hakami, Kumar Gular, Jaya Shanker Tedla, Mohammed Abohashrh

**Affiliations:** 1 Department of Medical Rehabilitation Sciences, College of Applied Medical Sciences, King Khalid University, Abha 62529, Saudi Arabia; 2 Department of Clinical Laboratory Sciences, College of Applied Medical Sciences, King Khalid University, Abha 62529, Saudi Arabia; 3 Department of Basic Medical Sciences, College of Applied Medical Sciences, King Khalid University, Abha 62529, Saudi Arabia

**Keywords:** bacterial culture, low-level laser therapy, gram stain, microwave diathermy, electrotherapy

## Abstract

*Background and Objectives*: The aim of this study was to compare the effects of low-level laser therapy and continuous microwave diathermy on the growth of Gram-negative and Gram-positive bacteria and to establish their efficacy as an alternative therapeutic modality. *Materials and methods*: Laser fluence of 13 Joules (J)/cm^2^, 18 J/cm^2^ and 30 J/cm^2^ were used against several bacterial strains. Microwave dosages of 25, 50 and 100 watts (W) were used, respectively. *Results*: A significant difference between the three groups was observed using repeated analysis of variance (RANOVA) (F value: 0.74, and *p* value: 0.001). The Greenhouse–Geisser correction (GG) revealed significant results for laser irradiation alone. However, effect size calculation showed effects with microwave diathermy as well as laser fluence. *Conclusions*: Low-level laser therapy appears to be an effective modality of treatment when compared with continuous microwave diathermy on the Gram-negative and the Gram-positive bacterial strains tested. Microwave diathermy revealed large and medium effects on the bacterial cell counts with dominant effects on Gram-negative strains.

## 1. Introduction

Antimicrobial drug therapy is frequently associated with the emergence of resistance resulting in morbidity and mortality worldwide [1]. The difficult-to-treat multidrug resistance most commonly leads to a state of economic stress on the healthcare providers that indulge them to use broad spectrum antibiotics which is often unwarranted [2].

There has been an exponential rise in hospital-acquired infections worldwide [3]. In the Arab world, including the Kingdom of Saudi Arabia, the prevalence of cases of multidrug resistant bacteria is on a rise [3,4]. As a result, non-healing pressure ulcers are a very common presentation among the patients suffering from chronic ailments admitted to the hospitals [5]. A study screening the incidence of pressure ulcers in patients with an average stay of 30 days in the hospital, found a high incidence of 39.3% of stage II–IV ulcers. The authors further recommended immediate strategies to control such a high incidence rate in the Kingdom [5].

Currently numerous treatment strategies are available to prevent the incidence of pressure ulcers among patients admitted to the hospitals but usually they fail to prevent the occurrence or re-occurrence of such pressure ulcers [6]. One reason could be its inefficiency to act against a wide range of organisms.

On the contrary, a systematic review analyzing the studies with low-level laser therapy (LLLT) with single wavelength laser probes (1: 904 nm vs. control, and 2: 940 nm vs. 808 nm vs. 658 nm), revealed that there was a significant difference observed with the use of a laser probe of 658 nm wavelength, whereas no supportive evidence was found for the laser probes using other wavelengths in the treatment of pressure ulcers [7].

In addition to lasers, another physical therapy modality, microwave and shortwave diathermy—which also operates in electromagnetic spectrum—are less explored and warrants extensive research on their beneficial effects in wound healing and antimicrobial activities [8]. In some studies, the researchers have also established the histomorphochemical effects of shortwave diathermy in animal models with better healing effects in the experimental group as compared to the controls [9]. Still, the effects of microwave diathermy in wound healing as a physical therapy modality is mostly unknown.

Microwave energy gets easily absorbed into the tissue as vibrational energy. Due to the dielectric nature of the tissue, this vibrational energy is converted into perceivable warmth in the tissue. The distribution of heat within the tissues is caused by the passage of microwaves, which in turn depends upon the properties of propagation and absorption of the irradiated tissue [10]. It is assumed that microwaves with the frequency of 2450 MHz penetrate 1.7 cm inside muscles and skin, and 11.2 cm inside adipose tissue and bone. Moreover, these values were increased to 3.04 cm and 17.7 cm, respectively, while using a frequency of 915 MHz [10].

On the contrary, a study on the evaluation of LLLT effects on wound healing concluded that LLLT can be safely applied on cutaneous wounds to accelerate the resolution of inflammation and healing; the researchers clearly outlined the selection of dose, duration and wavelength to be the primary factors in wound healing [11]. Various simulation models suggest that the depth of penetration of laser radiation at wavelengths of 630 nm to 1100 nm may vary, and can reach up to 50 mm [12]. In spite of that, the researchers failed to document its broad-spectrum effect against a wide variety of bacterial strains.

As it is clear from previous researches that LLLT and microwave are effective in causing healing, its antimicrobial effects against a wide range of organisms still lacks evidence. Hence the objective of the present study was to compare the effects of LLLT and microwave diathermy and to establish their efficacy as an alternative therapeutic modality to inhibit the growth of Gram-positive and Gram-negative bacteria in an in vitro model. To add further, there is a need to explore alternative therapies that can be easily engaged in the future in a clinical or hospital setting where cases of drug resistance are quite common.

## 2. Materials and Methods

Ethical approval was granted by the University Ethical Committee before the commencement of the study, with the approval number of REC#2017-03-10.

### 2.1. Bacterial Strains

Bacterial strains used in this study were the American Type Culture Collection (ATCC): *Pseudomonas aeruginosa* (ATCC 27853), *Escherichia coli* (ATCC 25922), *Enterococcus faecalis* (ATCC 29212), *Staphylococcus epidermidis* (ATCC 12228), *Streptococcus pyogenes* (ATCC 19615); *Shigella*, *Salmonella* sp., *Staphylococcus saprophyticus*, *Salmonella typhi*, *Staphylococcus epidermidis*, *Staphylococcus aureus* and *Klebsiella pneumonae*. All strains were cultured in Brain Heart Infusion (BHI) liquid broth at 37 °C. Bacteria were prepared in a suspension equivalent to 1 × 10^2^ colony forming units (cfu)/mL in BHI broth.

### 2.2. Laser Parameters

The sessions were conducted using a class 4 Laser M 1000 plus (Level-Laser Co., Moglano Veneto-Milano, Italy). The equipment comes with a pneumatic foot control switch, which can be used to turn the laser beam on and off. The laser parameters were as follows: The laser device was a semiconductor medium Ga-Al-As, generating a maximum power of 1 Watt continuous wave (CW) at 810 nm wavelength and a frequency of 500 Hz. The spot size of the laser beam was 0.5 cm^2^ with a duty cycle of 50% and voltage of 240 V. The fluence of the laser probe per point to irradiate Gram-positive and Gram-negative strains were 13 Joules/cm^2^ (J) for 36 s, 18 J/cm^2^ for 60 s and 30 J/cm^2^ for 80 s. The laser machine produced a sound beep when the treatment session ended. The therapist wore specific glasses as a protection from the laser beam. No complications were reported during the trial.

The accuracy of the output dosage of the laser machine was tested prior to irradiation using a specialized photodiode equipment (dosimeter). The in-built dosimeter was used to calibrate the probe before and during the treatment to ensure an even energy density distribution at all times of treatment.

### 2.3. Microwave Diathermy Parameters

The Radarmed 950+ device (Enraf nonius, Rotterdam, The Netherlands) was used to deliver microwave therapy. This device is usually used for heat therapy with the choice of both continuous and pulsed release modes for the waves. A continuous release of up to 250 Watt and a pulsed release with a maximum of 1500 Watt (W) can be chosen. This makes it possible to heat deeper tissue with a low dose. The machine provides easy switching between continuous and pulsed modes during treatment. The parameter settings remain visible on the display during the treatment sessions through an LCD display. A continuous mode with a frequency of 2450 MHz ± 50 MHz with the power of 25 W for 30 min, 50 W for 10 min and 100 W for 5 min were used.

### 2.4. Experimental Procedure

After each exposure, the total number of viable cells of each bacterial strain was counted as colony forming units (CFU) on BHI agar media incubated for 24 h at 37 °C. A laser beam was exposed on the 96-well plates containing 200 µL bacterial solution in each well followed by an exposure with microwave radiations. Samples were equally distributed in all microtiter wells. Following exposure, the samples were analyzed using serial dilution methods. All strains were treated with LLLT and microwave therapy for 3 days. The non-contact method was used for both microwave and laser irradiation of the samples to prevent soiling of the plate and probe surfaces.

### 2.5. Data Analysis

As the data was skewed, log transformation was applied for all the variables. The transformed units were then back-transformed by raising 10 elevated to the power of the number. The mean and standard deviation were reported as a measure of central tendency and dispersion of all continuous variables. The repeated-measures of analysis of variance (RANOVA) was used to evaluate the changes in the outcome measures for various dosages as assigned under the test group and the control group with measurements of the samples between the three groups. The probability value of *p* < 0.05 was considered statistically significant. As the Mauchly test was significant (*p* < 0.05), the Greenhouse–Geisser correction factor (GG) was used to interpret the results. The tests were performed using Statistical Package for the Social Sciences (SPSS) 20. Degrees of freedom (df 1, df 2), F values and *p* values were reported in RANOVA tests. Effect size of the various dosages on the samples were also calculated and quantified as described by Cohen et al. [13].

## 3. Results

The mean and standard deviation of the individual bacterial growth is shown in Table 1 for laser, microwave and the control group. Results interpretation was done using tests of within-subject effects which indicates whether there is an overall significant difference between the means at different time points. The tests of within-subjects for the main effects showed a *p* value less than 0.001, df (2, 4) and an F value of 7.04, indicating a significant difference between the three groups.

However, test of within-subject analysis for individual dosages using the Greenhouse–Geisser correction (GG) were as follows: The laser group with the fluence of 13 J/cm^2^ dosage had an F value of 7.5, ff value (1, 3) and *p* value of 0.07. Results with the fluence of 18 J/cm^2^ showed an F value of 10.27, df value of (1, 3), and *p* value of 0.05, whereas the fluence of 30 J/cm^2^ showed an F value of 10.35, df value of (2, 3) and *p* value less than 0.04. The individual analysis of dosages with microwave diathermy using 25 Watt had an F value of 1.9, df value of (1, 2) and *p* value of 0.30; for 50 Watts dosage an F value of 1.09, df value of (1, 2) and *p* value of 0.30 was found; and for 100 watts an F value of 1.74, a df value of (1, 2) and *p* value of 0.32 was found.

**Figure 1:**
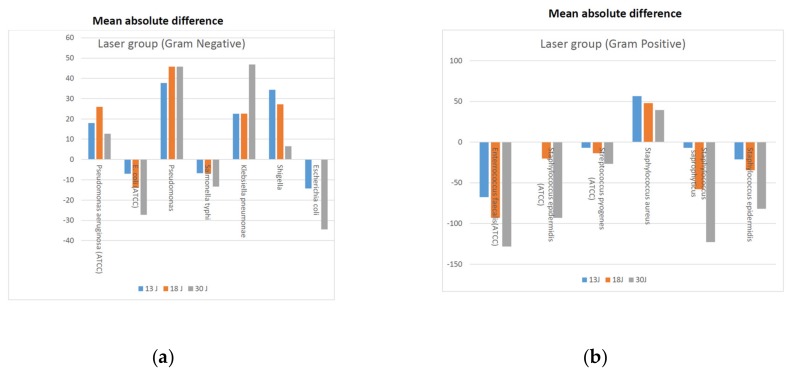
(**a**) Mean absolute difference post irradiation of Gram-negative strains in the laser group. (**b**) Mean absolute difference post irradiation of Gram-positive strains in the laser group.

**Figure 2:**
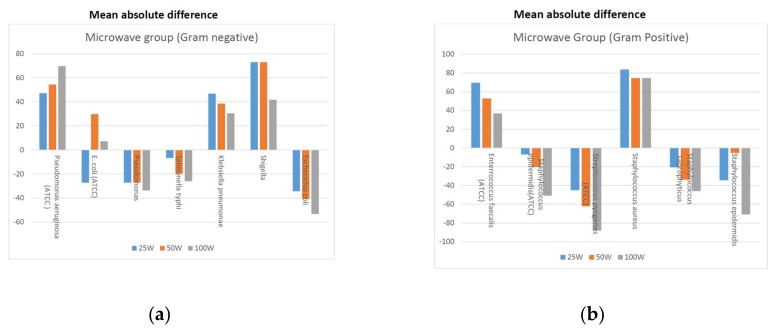
(**a**) Mean absolute difference post exposure of Gram-negative strains in the microwave diathermy group. (**b**) Mean absolute difference post exposure of Gram-positive strains in the microwave diathermy group.

Mean absolute difference was also calculated for Gram-negative and Gram-positive bacteria under laser and microwave groups, respectively (Figure 1a,b and Figure 2a,b). In the laser group the absolute mean difference revealed that the effect of dosages on the samples of Gram-negative strain of *E. coli* (ATCC 25922) and *S. typhi* showed reduction in counts, whereas greater mean difference was seen in Gram-positive strains in the samples of *E. faecalis* (ATCC 29212), *S. epidermidis* (ATCC 12228), *S. saprophyticus* and *S. epidermidis*. The reduction in bacterial counts were more prominent with the fluence of 18 and 30 J/cm^2^.

A reduction in cell counts was observed using microwave treatment with *E. coli* (ATCC 25922), *Pseudomonas* sp. and *S. typhi*. The same observation was noticed with the Gram-positive strains, namely *S. epidermidis* (ATCC 12228), *S. saprophyticus* and *S. pyogenes* (ATCC 19615). Moreover, highly noticeable reductions in the colony forming units were seen using 50 and 100 Watts.

However, it was also noted that three Gram-negative bacteria out of the seven tested species in the laser group showed changes in the cell count, whereas in the microwave group, four organisms showed a reduction in the cell count. Five out of six Gram-positive bacteria showed a reduced cell count with the laser irradiations as compared to the microwave group, where only four out of six organisms showed a reduced value of colony forming units.

The details of the effect size—which is considered as one of the most imperative indicators for clinical significance—was calculated for laser and microwave groups (Table 2) with the desired direction of treatment. The classification by Cohen et al. is as follows: <0.2 = trivial effect; 0.2–0.5 = small effect; 0.5–0.8 = moderate effect; >0.8 = large effect [13]. In the present study, negative effect size indicated a decrease in the bacterial cell count and vice versa.

## 4. Discussion

The present study scrutinizes the effect of various dosages of microwave and laser therapy on selected species of Gram-positive and Gram-negative bacteria. Laser and microwave are electrotherapeutic devices that are commonly used for musculoskeletal related problems. Laser has been used previously in wound healing with evidence-based productive effects [14,15,16,17]. On the contrary, microwave diathermy has been used in wound healing with significant epithelialization of wound area [18], but little is known about their effect on organisms.

In our investigation, we found that certain bacterial species presented a dose-specific sensitivity to exposure, depending on the energy delivered to the bacterial culture. When laser was irradiated on Gram-negative bacteria, it was observed that *E. coli* and *S. typhi* showed decreased cell count with the fluence of 13 J/cm^2^, 18 J/cm^2^ and 30 J/cm^2^, respectively (Table 1 and Table 2, and Figure 1 and Figure 2). Through this study the most efficacious fluence appears to be 30 Joules (Table 2). Out of the seven Gram-positive bacterial strains, three showed reduction in the CFU count, whereas five Gram-negative bacteria showed an obvious drop in the colony numbers counted. This finding is consistent with the Yuan et al. study. They found that the physiological function of the cells could be compromised with the laser probe at 532 nm until the physical disintegration of the cells [19].

Another study that used 904 nm wavelength laser and a fluence of 3 J/cm^2^ for seven days on Wistar rats did not alter the growth of *S. aureus* in vitro [20]. One of the postulated reasons could be that the fluence used for antibacterial effects in the study was too small to generate a noticeable effect. Though, similar findings were also observed in the present study where we found no effect of laser irradiation on the Gram-positive bacteria *S. aureus*. This clinically significant organism appears to be resistant to low-level laser irradiation dosages, an observation that has previously been reported [21]. Moreover, an effect can be seen with *S. aureus* irradiated with higher dosages, but the physiological adverse effects of higher dosages cannot be ignored in clinical settings.

In the microwave diathermy group, four Gram-negative bacteria and four Gram-positive bacteria showed a reduction in the cell count. It is possible that microwave could be more effective against Gram-negative strains as compared to LLLT. Though, the statistical methods revealed non-significant results, but when the effect size (a clinical significance measurement) was used, it showed conspicuous differences (Table 2 and Figure 2). Effect size usually mirrors the extent of the difference in the outcomes between the groups. A greater effect size indicates a larger difference between the experimental and the control group. Cohen’s effect may be positive or negative, indicating the course of the treatment [13,22]. In this study, the classification of Cohen et al. [13] was used to understand the clinical significance of the results (Table 2).

An abnormal increase in cell count was also observed in the study. Though the mechanism responsible for the same is currently not well understood, there is a need for prior wound culture of the infected wound area before they are exposed to any kind of electrotherapeutic modalities. The present findings were also reported by Nussbaum et al. They found an abnormal increase in the cell count of *E. coli*, *P. aeruginosa* and *S. aureus* post irradiation in in vitro samples [15,21].

Some novel studies recommended the use of blue laser light on *S. aureus* in in vitro models, and found strong evidence of early antimicrobial activity [23]. However, such studies are subjected to further preclinical trials before they can be essentially implemented.

Microwave diathermy has both thermal and non-thermal effects [10]. The non-thermal effects have the capability to alter cell membrane activity, causing resolution of inflammations by vasodilation. This could be the therapy of choice by irradiating chronic multidrug resistant open wounds. Microwave seems to be more effective against Gram-negative bacteria than Gram-positive bacteria. These results should be taken with precaution as some bacterial strains showed a tendency to multiply exponentially post exposure.

Microwave diathermy has recently demonstrated that low-dose microwave may augment healing of fractures [24]. Though it has been established that microwave has a sterilizing ability on microbial cells in vitro [25,26], the effect on wound healing should be determined clinically [27]. Some researchers have also hypothesized that low frequency dosages may have a stimulatory effect on certain bacterial strains [27]. This finding is in line with the results of the present study. On the contrary, a study which has used continuous mode (CM) of microwave diathermy on 71 patients reported beneficial effects of low-intensity CM on the treatment of post-operative purulent wounds caused by pyogenic *S. aureus* after abdominal surgery [18]. Such findings are subjected to extensive exploration in preclinical research before the results can be applied clinically.

Moreover, there could be various postulated reasons for the observed variations in the study, which may be due to the mode of application or fluence over the isolated colonies without contact of the probe, which may have caused energy loss during irradiation. As irradiation decreases due to the inverse square law, the intensity of the incident radiation is inversely proportional to the square of the distance between the source and the surface causing increased reflection [15].

Another postulated factor may be single wavelength parameters and the difference in cell structure and virulence factor [20]. Usually, the bactericidal effect of LLLT is due to the direct effect on the bacterial membrane due to absorption of photons by endogenous chromophores, with simultaneous production of highly reactive and cytotoxic molecules which causes disruption of the membrane and resulting in bacterial killing. Whereas some organisms, after irradiation by laser, tend to stimulate or proliferate which is related to changes generated by increased energy intake provided by the radiations in the respiratory chain of the bacteria [28]. Nussbaum et al. had also proposed that the photobiological response of organism to exposure to monochromatic light depends directly on irradiation parameters and culture conditions [15].

There were a few limitations in the study, firstly, we were not able to evaluate the effects of single versus multiple exposure of the modalities to the cell culture, and secondly the temperature of the culture medium pre-post irradiation was not measured.

## 5. Conclusions

Low-level laser therapy using an 810 nm wavelength probe appears to be an effective modality of treatment when compared with continuous microwave diathermy on Gram-negative and Gram-positive bacteria. Though, the effect size calculation of the microwave diathermy group showed large and medium effects on the bacterial cell counts with a dominant effect on Gram-negative strains. It is also recommended that the potential effects of exposure by electrotherapeutic modalities on the bacterial species should be monitored prior with wound cultures. Moreover, these modalities provide non-invasive treatment which can be successfully used for chronic inflammatory and infectious wounds and other skin diseases. One of the strengths of these modalities are represented by their usage among patients exhibiting drug resistance against a wide range of bacteria’s, and the chance of relatively little adverse effects add to its high safety profile for usage in elderly and/or immuno-depressed patients. 

## Figures and Tables

**Table 1:** Treated and control groups results measured as CFU/mL with their mean, standard deviation and confidence interval.

Organisms	Origin							
		Treated (Cfu/mL)						
		Control (cfu/mL)	Laser Group			Microwave		
Energy		No Treatment	13 J/cm^2^	18 J/cm^2^	30 J/cm^2^	25 W	50 W	100 W
*Pseudomonas aeruginosa* (ATCC 27853)	R	269.15 ± 3.02 (812.8–89.1)	251.19 ± 3.48 (871–72.4)	295.12 ± 3.39 (1000–87)	281.84 ± 3.39 (977.2–81.3)	316.23 ± 3.39 (1071.5–93.3)	323.6 ± 3.16 (1023.3–102.3)	338.84 ± 3.16 (1023.3–977.2)
*E. coli* (ATCC 25922)	R	309.03 ± 3.02 (933.3–102.3)	302 ± 3.55 (1071.5–85.1)	295.12 ± 3.63 (1071.5–81.3)	281.84 ± 0.57 (1047.1–85.1)	281.84 ± 3.72 (1096.5–104.7)	338.84 ± 3.24 (1096.5–104.7)	316.23 ± 3.39 (1071.5–93.3)
*Pseudomonas* sp.	C	309.03 ± 3.16 (977.2–97.7)	346.74 ± 3.72 (1288.3–93.3)	354.8 ± 3.47 (1230.3–10.2)	354.8 ± 3.31 (1174.9–10.7)	281.84 ± 3.31 (933.3–85.1)	281.84 ± 3.89 (1096.5–72.4)	275.42 ± 3.89 (1071.5–70.8)
*Salmonella typhi*	C	295.12 ± 2.88 (851.1–102.3)	288.4 ± 3.31 (955–87.1)	288.4 ± 3.31 (955–87.1)	281.84 ± 3.31 (933.3–85.1)	288.4 ± 3.39 (955–87.1)	275.43 ± 3.39 (933.3–81.3)	269.15 ± 3.39 (912–79.4)
*Klebsiella pneumonae*	C	316.23 ± 2.82 (891.3–112.2)	338.8 ± 3.39 (1148.2–100)	338.84 ± 3.31 (1122–102.3)	346.74 ± 3.16 (1096.5–109.7)	363.08 ± 3.09 (1122–117.5)	354.81 ± 3.16 (1122–112.2)	346.74 ± 3.16 (1096.5–109.7)
*Shigella* sp.	C	281.84 ± 3.02 (851.14–93.3)	316.23 ± 3.47 (1047.1–91.2)	309.03 ± 3.47 (1071.5–89.1)	288.4 ± 3.72 (1071.5–77.6)	354.81 ± 2.69 (955–131.8)	354.81 ± 2.69 (955–131.8)	323.59 ± 3.02 (977.2–107.2)
*Escherichia coli*	C	316.23 ± 2.95 (933.3–107.2)	295.12 ± 3.46 (1023.2–85.1)	316.2 ± 3.47 (1096.5–91.2)	281.84 ± 3.63 (1023.3–77.63)	281.84 ± 3.80 (1071.5–74.1)	275.42 ± 3.89 (1071.5–70.8)	263.03 ± 4.07 (1071.5–64.6)
*Enterococcus faecalis* (ATCC 29212)	R	302 ± 2.69 (812.8–112.2)	234.42 ± 2.88 (676.08–81.28)	208.93 ± 2.82 (588.8–74.13)	173.78 ± 2.88 (501.2–60.3)	371.53 ± 2.46 (1148.2– 151.4)	354.81 ± 2.51 (912–141.3)	338.84 ± 2.57 (871–131.8)
*Staphylococcus epidermidis* (ATCC 12228)	R	302 ± 2.95 (891.3–102.3)	302 ± 3.47 (1023.3–85.1)	281.84 ± 3.63 (1023.3–77.6)	208.93 ± 4.07 (851.1–51.3)	295.12 ± 3.47 (1023.3–85.1)	281.84 ± 3.63 (1023.3–77.6)	251.19 ± 4.17 (1047.1–60.3)
*Streptococcus pyogenes* (ATCC 19615)	R	302 ± 3.09 (933.3–97.7)	295.12 ± 3.55 (1047.1–83.2)	288.4 ± 3.63 (1047.1–79.4)	275.42 ± 3.8 (1047.1–72.4)	257.04 ± 4.37 (1122–58.9)	239.88 ± 5.62 (1174.9–38)	213.8 ± 5.62 (1202.26–38)
*Staphylococcus aureus*	C	323.59 ± 2.95 (954.99–109.7)	380.19 ± 3.09 (1174.9–123.03)	371.54 ± 3.09 (1148.2–120.2)	363.08 ± 3.16 (1148.2–114.8)	407.38 ± 2.69 (1096.48–151.4)	398.11 ± 2.69 (1071.52–147.9)	398.11 ± 2.69 (1071.52–147.9)
*Staphylococcus saprophyticus*	C	309.02 ± 2.95 (912.01–104.7)	302 ± 3.39 (1023.3–89.13)	251.19 ± 3.55 (891.3–70.8)	186.21 ± 3.31 (616.6–56.2)	288.4 ± 3.47 (1000–287.9)	275.42 ± 3.63 (1000–75.9)	263.03 ± 3.89 (1023.29–1.8)
*Staphylococcus epidermidis*	C	316.23 ± 2.88 (912–109.7)	295.12 ± 3.47 (1023.3–85.1)	281.84 ± 3.55 (1000–79.4)	234.44 ± 3.88 (891.3–61.7)	281.84 ± 3.63 (1023.3–77)	263.03 ± 3.63 (1023.3–70.8)	245.47 ± 4.27 (1047.1–57.5)

R—Reference strain (Wild type strain), C—Clinical strains were isolated from the patients, Confidence Interval (CI) at 95%, Cfu/mL—Colony-forming units per milliliter.

**Table 2:** Calculation of effect size (r) and its interpretations post irradiation with different dosages.

Organisms						
	Treated (cfu/mL)					
	Laser Group (R Value)			Microwave		
Energy	13 J	18 J	30 J	25 W	50 W	100 W
***Gram-negative strains***						
*Pseudomonas aeruginosa* (ATCC 27853)	−0.94 * (LE)	+0.97 (LE)	+0.89 (LE)	+0.99 (LE)	+0.99 (LE)	+1 (LE)
*E. coli* (ATCC 25922)	−0.73 * (ME)	−0.90 * (LE)	−0.99 * (LE)	−0.97 * (LE)	+0.98 (LE)	+0.75 (ME)
*Pseudomonas* sp.	+0.98 (LE)	+0.99 (LE)	+0.99 (LE)	−0.97 * (LE)	−0.97 * (LE)	−0.98 * (LE)
*Salmonella typhi*	−0.74 * (ME)	−0.74 * (ME)	−0.91 * (LE)	−0.73 * (ME)	−0.95 * (LE)	−0.97 * (LE)
*Klebsiella pneumonae*	+0.96 (LE)	+0.97 (LE)	+0.98 (LE)	+0.99 (LE)	+0.99 (LE)	+0.98 (LE)
*Shigella* sp.	+0.98 (LE)	+0.97 (LE)	+0.70 (LE)	+1 (LE)	+1 (LE)	+0.99 (LE)
*Escherichia coli*	−0.96 * (LE)	−0.01 * (TE)	−0.98 * (LE)	−0.98 * (LE)	−0.99 * (LE)	−0.99 * (LE)
***Gram-positive strains***						
*Enterococcus faecalis* (ATCC 29212)	−1 * (LE)	+0.92 * (LE)	−1 * (LE)	+1 (LE)	+1 (LE)	+0.99 (LE)
*Staphylococcus epidermidis* (ATCC 12228)	0 (TE)	−0.95 * (LE)	−1 * (LE)	−0.73 * (ME)	−0.95 * (LE)	−0.99 * (LE)
*Streptococcus pyogenes* (ATCC 19615)	−0.72 * (ME)	−0.90 * (LE)	−0.97 * (LE)	−0.99 * (LE)	−0.9 * (LE)	−1 * (LE)
*Staphylococcus aureus*	+0.99 (LE)	+0.99 (LE)	+0.99 (LE)	+1 (LE)	+1 (LE)	+1 (LE)
*Staphylococcus saprophyticus*	−0.74 * (ME)	−0.99 * (LE)	−1 * (LE)	−0.96 * (LE)	−0.98 * (LE)	−0.99 * (LE)
*Staphylococcus epidermidis*	−0.96 * (LE)	−0.98 * (LE)	−1 * (LE)	−0.98 * (LE)	−0.99 * (LE)	−1 * (LE)

<0.2 = trivial effect (TE); 0.2–0.5 = small effect (SE); 0.5–0.8 = moderate effect (ME); >0.8 = large effect (LE). “−” Signifies reduction in bacterial cell count, “+” signifies increase in bacterial cell count * in accordance with the direction of treatment.
